# The use of HCT and/or ACE inhibitors significantly increases the risk of non-melanotic skin cancer in the periocular region

**DOI:** 10.1007/s00417-022-05576-y

**Published:** 2022-02-17

**Authors:** Juliane Mehlan, Julian Ueberschaar, K. Hagenström, C. Garbe, M. S. Spitzer, V. Druchkiv, F. Schuettauf

**Affiliations:** 1 Department of Ophthalmology, Medical Center Hamburg-Eppendorf (UKE), Martinistraße 52, 20246 Hamburg, Germany; 2 Research in Dermatology and Nursing (IVDP), Institute for Health Services, UKE, Martinistraße 52, 20246 Hamburg, Germany

**Keywords:** Hypertension, Skin cancer, Basal cell carcinoma, Photosensitization, ACE inhibitors, Hydrochlorothiazide

## Abstract

**Background/Aims:**

To investigate a possible association between the use of hydrochlorothiazide (HCT) and/or angiotensin-converting enzyme inhibitors (ACE inhibitors) and the occurrence of periocular non-melanoma skin cancer.

**Methods:**

The files of 929 patients from the University Medical Center Hamburg-Eppendorf who were surgically treated for suspected periocular malignancy were evaluated retrospectively regarding the occurrence of non-melanoma skin cancer and concomitant medication. To be able to put the data in an overall context, we also analyzed age-matched routine data of the DAK-Gesundheit (DAK-G), a nationwide operating German health insurance company.

**Results:**

Of the 929 patient records examined, who underwent surgical excision for suspected non-melanotic malignancy, non-melanocytic skin cancer could actually be determined by histology in 199 patients. In total, 176 patients (103 women, 72 men) had a basal cell carcinoma and 23 patients (16 women, 7 men) suffered from squamous cell carcinoma. The rate of intake of HCT or ACE inhibitors in our patient collective with non-melanotic skin cancer is significantly higher than in the general age-matched population (ORACE: 2.51, *p* < 0.001; ORHCT: 7.24, *p* < 0.001, ORBOTH: 4.61, *p* < 0.001).

**Conclusion:**

The rate of intake of HCT or ACE inhibitors is significantly higher in our patient collective with non-melanotic skin cancer compared to the group from the age-matched general population (DAK insured (*p* < 0.001)) compared to the routine data of the DAK-G. This leads us to the conclusion that taking the medication is associated with an increased risk for non-melanotic skin cancer. We recommend regular skin cancer screening, moderate ordination of photosensitizing medication, but above all comprehensive clarification of possible risks.
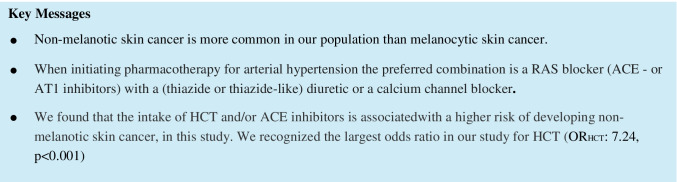

## Introduction

Non-melanotic skin cancer is more common in our population than melanocytic skin cancer. Overall, the mortality in non-melanoma skin cancer is lower. It is estimated that around 213,000 patients in Germany develop non-melanoma skin cancer every year. In the annual report of 2016, there were 77% basal cell carcinoma and 22% squamous cell carcinoma. The mortality of non-melanotic skin cancer in the same year is 750 patients per year [1].

In July 2008, skin cancer screening was introduced in Germany. This enables insured persons over the age of 35 to have a comprehensive examination every 2 years. Examinations usually take place at the family doctor or dermatologist. Incidence rates have suddenly risen since introduction—especially at early stages of the disease [1]. This can be demonstrated for both—melanotic and non-melanotic skin cancer. In a report on cancer events published by the Robert Koch Institute 2016, no decline in advanced tumor stages of melanocytic skin cancer was discernible until 2013 [1].

When initiating pharmacotherapy for arterial hypertension, the guideline “Management of arterial hypertension (version 2018)” of the German Society for Cardiology recommends a double combination, preferably as an SPC (single-pill combination). The preferred combination is a RAS blocker (ACE—or AT1 inhibitors) with a (thiazide or thiazide-like) diuretic or a calcium channel blocker [2].

Thiazide diuretics have been prescribed since the late 1950s and ACE inhibitors since the 1980s. They are among the most commonly prescribed drugs. The current combination preparations are available as generics and are usually taken once a day and permanently. The pack size N3 usually contains 100 tablets and is accordingly prescribed quarterly.

A study of Pedersen et al., carried out by the Danish Cancer Registry, proofed that the intake of high-dose hydrochlorothiazide (HCT) (cumulative dose 50 g) leads to an odds ratio of 1.29 for basal cell carcinoma and 3.98 for squamous cell carcinoma. The effect was described as dose-dependent [3].

Subsequently, at the end of 2018, in coordination with the European Medicines Agency (EMA) and the Federal Institute for Drugs and Medical Devices (BfArM),

a red hand letter was issued, explaining the connection between increasing doses of hydrochlorothiazide and the development of non-melanoma skin cancer.

Taking this into account, the question arises whether long-term medication with HCT or ACE inhibitors has an influence on the development of non-melanotic periocular skin cancer due to the photosensitizing effect and whether additional patient education and, in particular, interdisciplinary communication is necessary.

## Materials and methods

### Data source and study population

We evaluated the files of 929 patients who were operated at the Hamburg-Eppendorf Clinic between 2013 and 2018 on suspicion of an eyelid tumor. The evaluation was made pseudonymized and anonymized. Our evaluation included patients who had pre-operatively received antihypertensive therapy with ACE inhibitors and/or diuretic premedication (such as HCT) with hydrochlorothiazide as long-term therapy.

In addition, it was documented whether further suspicious skin lesions were documented during the dermatological screening.

We also analyzed the data of insured persons from DAK-Gesundheit (DAK-G). These routine data cover a 40% representative sample (2.4 million) of all insured persons of the DAK-G. We considered the period from December 31, 2010 until December 31, 2015. Statutory health insurance is firmly anchored in the German health system: Around 90% of the German population (around 73 million) are insured with one of the 105 statutory health insurance companies (in 2020). The remaining 10% are privately insured (The National Association of Statutory Health Insurance Funds (2020)). These data contain all billing-relevant information from the outpatient and inpatient sector, including outpatient-prescribed drugs (based on the Anatomical Therapeutic Chemical (ATC) Classification), coded diagnoses (according to the German modification of the International Classification of Diseases (ICD-10-GM)), and outpatient and inpatient operations and procedures (according to Classification for Operations and Procedures (OPS)). The ICD-10-GM is a translation of the ICD-10-WHO from the BfArM into German. The insured person’s master data contains socio-demographic information on age and sex, as well as the beginning and end of the insured time. All health service areas are linked with each other via a pseudonym and presented as aggregated data (Busse et al.; 2014). The descriptive analyses showed the prevalence of non-melanoma skin cancer (ICD-10-GM C44) and the prescription rate of insured persons with at least one prescription of HCT and/or ACE inhibitors for the year 2010 (ATC codes: hydrochlorothiazide C03AA03; ACE inhibitor C09A). A prevalent case was defined as an insurant with a reliable outpatient or main or secondary inpatient diagnosis of non-melanoma skin cancer in 2010. To validate the skin cancer diagnosis, the insured had to have an excision in the quarter of diagnosis or one quarter before.

This can exclude an overestimation of the skin cancer diagnosis. In addition, it was analyzed how many insureds had a surgery in the lid area or a skin cancer diagnosis (with excision) in 2015 and how many of these insureds received at least one prescription of the relevant drugs within 5 years prior to the diagnosis. For the same insured cohort, the consistent prescription rates of the relevant drugs within 5 years prior to the diagnosis were recorded. Consistent was defined as at least one prescription per year.

The evaluations were carried out on a quarterly basis.

### Statistical analyses

The annual prevalence was presented as crude and standardized percentage rate for 2010. Insured persons who met the case definition comprised the numerator for the prevalence estimation. The denominator was constituted from all insured persons in the sample. In addition, the prevalence estimates were standardized by age and sex to the German population, as of December 31, 2012 (direct standardization) [4], and extrapolated to the German population, as of December 31, 2010 [4]. The age was calculated based on year of disease, as of December 31, 2010, and year of birth.

The drug supply was presented as percentage rate for 2010. Insured persons who met the case definition comprised the numerator for the drug supply estimation. The denominator was constituted from all insured persons in the sample.

The analyses were performed according to current methodological standards [5] and by using SAS 9.4 (SAS Institute, Cary, NC, USA). The analysis of our data and its comparison to the data of DAK was performed with R Core Team.

## Results

The results of the retrospective analysis are presented to investigate the declaration of the recently issued *Direct Healthcare Professional Communication* (“Dear Doctor Letter”). This puts the premedication with hydrochlorothiazide in connection with the increased incidence of basal cell carcinomas and squamous cell carcinomas due to the photosensitizing effect. We screened all patient files of eyelid surgery for tumor excision, which were carried out in the years 2013–2018 at the university hospital.

**Fig. 1:**
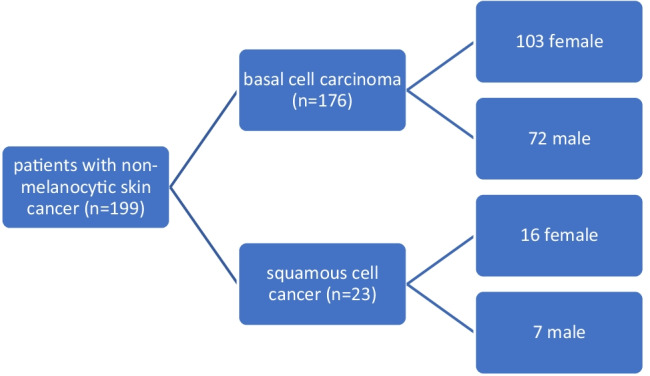
General information on patient population

In total, 199 patients of the 929 patients whose files we examined suffered from non-melanocytic skin cancer. Then, 176 patients (103 female, 72 male) had a basal cell carcinoma and 23 patients (16 female, 7 male) had a squamous cell carcinoma. The mean age of the patients was 75.7 years (SD 11.7 years; range 34–95 years). In the other 730 cases, no malignant tumor could be found on histological examination. In our sample, about 60% of the patients with non-melanotic skin cancer were women (Fig. 1).

Forty-three (21.7%) of the patients with basal cell carcinoma, had pre-medication with ACE inhibitors, 17 (8.6%) had pre-medication with HCT, and 12 (6.1%) of the patients were taking both drugs. Thus, we were able to prove a positive medication history in 64 (36.4%) of the basal cell patients.

**Fig. 2:**
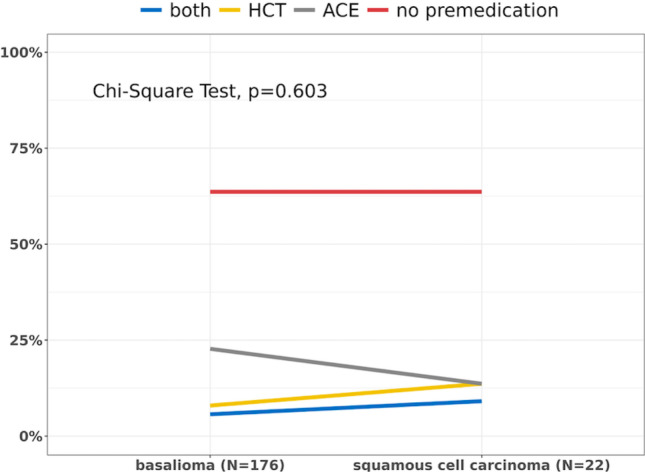
Distribution of pre-medication in non-melanoma skin cancer

Three (13.6%) of the patients with squamous cell carcinoma had pre-medication with ACE inhibitors, 3 (13.6%) had pre-medication with HCT, and 2 (9.1%) of the patients were taking both drugs. Thus, we were able to demonstrate a positive medication history in 8 (36.4%) of the patients with squamous cell carcinoma. Unfortunately, we do not have complete drug information for one of the 23 patients with squamous cell carcinoma. Therefore, the case in the graphic has been removed and this explains the number of 22 patients. The study design (anonymized and pseudonymized) unfortunately does not allow us to trace back or complete it. We have therefore excluded this case from further statistical processing of the data. The results are shown graphically in Fig. 2.

### Administrative data

In 2010, 2,319,584 people were insured throughout the DAK-G sample. Approximately two-thirds of the insured (62%) were female. Compared to our sample, there are no significant differences regarding the distribution of the sexes (*p* = 0.571).

### Prevalence of skin cancer and the use of HCT and/or ACE inhibitors

**Fig. 3:**
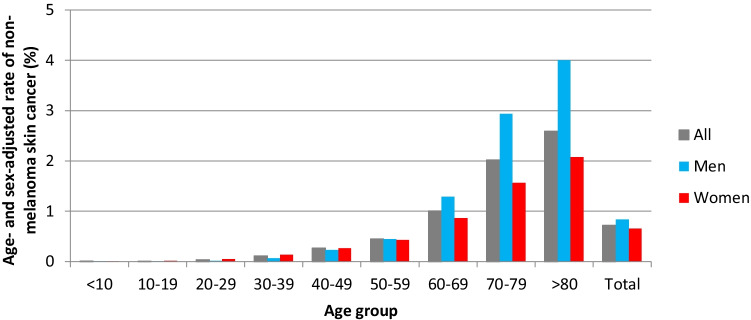
Age- and sex-adjusted prevalence rate of non-melanoma skin cancer in 2010 (*N* = 2,319,584)

In 2010, 20,607 of 2,319,584 insured people were diagnosed with a non-melanoma skin cancer (diagnosed by an operation) (Fig. 3). This value corresponds to a crude prevalence rate of 0.89% and an age- and sex-adjusted rate of 0.72%. The extrapolation to the German population in 2010 shows that 587,051 people suffered from non-melanoma skin cancer (diagnosed by an operation).

Female had a prevalence rate of 0.79% (age- and sex-adjusted rate: 0.65%), increasing with age. Male showed a crude prevalence rate of 1.04% (age- and sex-adjusted rate: 0.84%), also increasing with age and exceeding the quota for women aged 50 and over. The mean age was 71.38 years (SD 11.71 years; range 3–103 years).

**Fig. 4:**
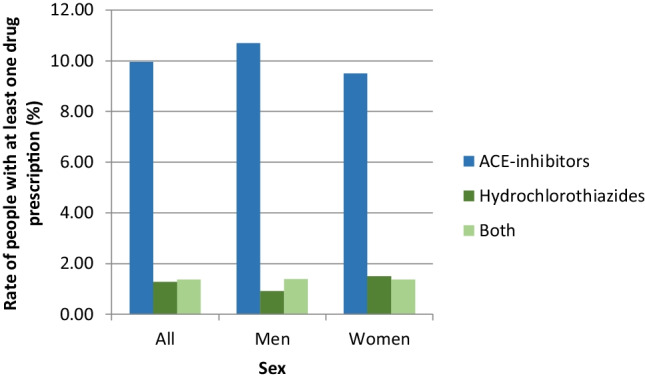
Rate of people with at least one drug prescription in 2010 by sex and drug group (*N* = 2,319,584)

In total, 292,733 people had at least one drug prescription of the relevant drugs in 2010; this corresponds to a rate of 12.62% (Fig. 4). Overall, more men than women take ACE inhibitors (10.69% vs. 9.50%). Otherwise, more women than men received hydrochlorothiazides (1.51% vs. 0.92%). Regarding both preparations, there were no sex differences (1.38%).

**Fig. 5:**
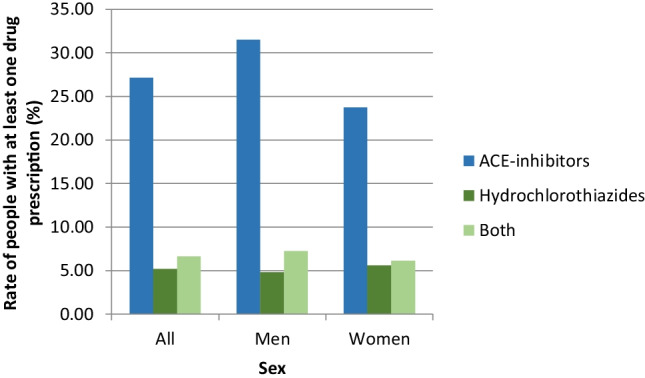
Rate of people with at least one drug prescription within 5 years before non-melanoma skin cancer diagnosis (with excision) in 2015 by sex and drug group (*N* = 29,706)

In total, 29,706 people (1.64%) got at least one prescription before non-melanoma skin cancer diagnosis or a surgery in the periocular area (Fig. 5). The mean age of these patients was 73.66 (SD 11.75 years; range 6–105 years). Then, 39.01% (11,588) of this cohort got at least one prescription of the relevant drugs between 2021 and 2015. 27.15% of the insured received at least one prescription of ACE inhibitors within 5 years prior to the diagnosis of non-melanoma skin cancer or an operation in the periocular region. 5.23% got at least one prescription of hydrochlorothiazides and 6.63% got both, ACE inhibitors and hydrochlorothiazides. Interestingly, men showed a higher ACE inhibitor intake than women (31.50% vs. 23.74%) and a slightly lower intake in hydrochlorothiazides than women (4.80% vs. 5.57%).

In our sample, there are significant differences between males and females, as well (*p* = 0.018).

Males take more ACE inhibitors (32.9% vs. 14.3%, *p* = 0.003) and less HCT (6.3% vs. 10.1%, *p* = 0.443), and men take the combination of the two products less often (3.8% vs. 7.6%, *p* = 0.369).

An age-adjusted comparison of the data from our patient population with the comparison group from the general population (DAK insured), with at least one drug prescription within 5 years before non-melanoma skin cancer diagnosis (with excision), showed that there was no significant difference between the two groups (no statistically significant difference found, *p* = 0.083).

Furthermore, the rate of intake of HCT or ACE inhibitors in the comparison group from the general population (DAK insured) (Fig. 4) is significantly lower than in our patient collective with non-melanotic skin cancer (*p* < 0.001).

From both analyses, it can be concluded that the risk of non-melanoma skin cancer increases when taking the medication.

We also calculated the odds ratio. In our patient collective, the risk of developing non-melanoma skin cancer is 2.5 times higher than in the comparison group of the DAK-G (ORACE: 2.51, *p* < 0.001). This difference is statistically significant.

The risk of taking HCT in our patient group is 7.24 times higher than in the comparison group (ORHCT: 7.24, *p* < 0.001). This is also significant.

When taking both preparations, the risk of our patients was 4.61 times higher than with the normal population (ORBOTH: 4.61, *p* < 0.001).

## Discussion

This study shows that 36.4% of patients with histologically proven basal cell carcinoma and 36.4% of patients with squamous cell carcinoma have pre-medication with HCT and/or ACE inhibitors. A comparison—age group adjusted—of the data of our patient population with the comparison group from the general population (DAK-insured persons) with at least one drug prescription before the diagnosis of non-melanoma skin cancer showed no significant difference between the two groups (*p* = 0.083). Thus, it can be concluded that the study group corresponds to the average of the population and that conclusions are possible, if the rates of non-melanoma skin cancer under the above-mentioned medication within the population of our study agree with the comparison group of the general population.

These rates of intake are significantly higher than those of the general population, *p* < 0.001, and from the population with continuous use of drugs before surgery, *p* < 0.001.

The results of this study therefore support the results of Pedersen et al. on the potential connection between HCT use and the occurrence of basal cell carcinomas and squamous cell carcinomas [3].

It can be postulated that not only taking hydrochlorothiazide but also taking ACE inhibitors seems to favor the development of non-melanoma skin cancer. This is due to the photosensitizing effect of the drugs. The effect is based on the supply of exogenous chromophores (in this case ACE inhibitors or HCT), which mostly absorb photons from sunlight. This leads to their activation and chemical reactions. The resulting free radicals usually cause direct cell damage, but can also lead to the oxidation of cellular lipids via the formation of reactive oxygen species.

Hypersensitivity reactions, on the other hand, are mostly based on haptenization.

Photoallergic reactions are to be separated from phototoxic reactions [6]. Around 300 different photosensitizing agents are known in Germany. For this reason, patients with the above-mentioned long-term medication require strict dermatological and ophthalmological follow-up examinations, and patient education should also be intensified.

In the evaluation of the registry data from Denmark, a significant increase in the rates of non-melanoma skin cancer could only be demonstrated for HCT [3]. This could be explained by the prescribing behavior, since HCT is prescribed very often in the sense of a first-line therapy. In some data, HCT is also mentioned as the most frequently prescribed substance [7].

In our study, 22.7% of the patients with registered basal cell carcinomas took ACE inhibitors. In contrast, only 8.0% of the patients who had a biopsy-proven basal cell carcinoma of the periocular region indicated that they were medicated with HCT. The variance to the data from Denmark could be explained by the fact that in Germany, significantly more patients take ACE inhibitors (around 19% of the population) than HCT (around 6% of the population) [8]. At the end of 2018, European Medicines Agency (EMA) and the Institute for Drugs and Medical Devices (BfArM) issued a red hand letter that highlighted the connection between increasing doses of hydrochlorothiazide and the development of non-melanoma skin cancer.

At that time, regular skin cancer screening was also recommended for patients taking long-term therapy. Any changes that suspect malignancy should also be surgically removed and biopsy secured.

As a primary, preventive measure, adequate sun protection is also important. Patients should be informed in detail about this in order to reduce the general risk of skin cancer [9]. According to the S3 guideline for skin cancer prevention, the avoidance of excessive exposure to sunlight, suitable clothing, and sunscreen are the most important preventive measures (guideline program oncology; 2014). In the etiology of squamous cell carcinoma, the total UV light exposure plays a decisive role, while the risk for the development of a basal cell carcinoma is particularly associated with sunburn [10].

The prevention and the consideration of risk factors that lead to the development of melanocytic and also non-melanocytic human cancer play an increasingly important role. Besides, one must keep in mind that sport and leisure behavior have changed and there is an increasing trend towards outdoor activities, but also towards travelling to southern regions for several decades. This underlines once again the importance of comprehensive education and prevention.

Women had an age- and gender-adjusted rate of 0.65% for non-melanoma skin cancer, and men had an age- and gender-adjusted rate of 0.84%. In contrast, women take ACE inhibitors (10.69% vs. 9.50%) and HCT (1.51% vs. 0.92%) more often than men. However, there were no gender differences between the two preparations (1.38%).

Therefore, two things could serve as a possible explanation. First, the drugs are not the sole cause of the development of non-melanoma skin cancer. Exposure to sunlight or tobacco smoke would also have to be included in the declaration for the analysis of the data on the general population. Second, it cannot be ruled out that there is a certain selection aspect (regarding the population), as this concerns the DAK-insured persons. Follow-up studies would have to explain and justify this aspect in more detail.

As a limitation of our study, it should be clearly stated that a dose-dependency cannot be proven. This is due to the retrospective nature of our study. Further longitudinal surveys are planned in the further course, considering the dose dependency, as well as preventive measures that have already been applied. Also we have to add that the lack of information on UV exposure and tobacco smoking is a limitation of our study. We are taking this into account for follow-up studies.

The increased risk of lip cancer after taking HCT has long been proven [11]. Yet further analyses must certainly clarify to what extent the risk of non-melanotic skin cancer is influenced in other parts of the body and how high the odds ratio is. It will be of particular interest here to what extent the risk increases in areas of skin that are less exposed to sunlight.

About 90% of the German population have statutory health insurance. These data are not only of numerical importance, but also provide scientific added value in relation to the population as well as in the sectoral and longitudinal availability of the data [12].

The routine data were not collected for research purposes. For this reason, it is only possible to depict the health care situation of insured persons who have actually made use of the health care system. The real disease rates may therefore exceed the results. Factors such as socio-economic status, clinical parameters or individual health behavior cannot be mapped using SHI data and may cause differences in comparison groups [12]. Due to the lack of clinical data, no distinction can be made between basal cell carcinoma and squamous cell carcinoma in the diagnosis of non-melanoma skin cancer.

In summary, we recommend a moderate ordination of photosensitizing medication. Furthermore, the patients should be informed about the possible risks and receive regular dermatological and ophthalmological controls.

Comprehensive clarification of possible risks is one of the most important points.

A change in therapy or termination is recommended if the patient has already been diagnosed with non-melanoma skin cancer. Follow-up studies must help to clarify the extent of other factors, such as the influence of sunlight or nicotine.
